# Adjuncts and alternatives in the time of antibiotic resistance and in‐feed antibiotic bans

**DOI:** 10.1111/1751-7915.12730

**Published:** 2017-06-22

**Authors:** Harald Brüssow

**Affiliations:** ^1^ Department of Gut EcologyHost‐Microbe Interaction GroupNestlé Research CenterLausanneSwitzerland

At first glance, it might surprise when an editorial series in a scientific journal asks how science will help to create companies and jobs. However, a recent ‘Nature’ editorial has encouraged ‘research leaders in the United States and elsewhere to address the needs and employment prospects of taxpayers who have seen little benefit from scientific advances’ (Editorial, 2017). Therefore, mundane as it sounds, the question of whether microbiome research can be a source of new enterprise and job creation matters not only for microbiologists with respect to work opportunities (this might amount to preaching the already converted), but also to a wider audience, which needs to be convinced that science can better the lot of society. In the current editorial, I want to explore where microbiome research can contribute in finding and developing alternatives and adjuncts to antibiotics.

The public health incentive for alternatives is indeed high: the Centers for Disease Control and Prevention (CDC) calculated 23 000 deaths per year in the USA attributed to infections with antibiotic‐resistant bacterial pathogens (https://www.cdc.gov/drugresistance/), and a UK government report estimated that antibiotic‐resistant infections kill 700 000 people each year worldwide (https://amr-review.org/). An increase to worldwide 10 million deaths per year is projected for 2050 if antibiotic resistance continues unabated (https://amr-review.org/). Therefore, curbing antibiotic resistance is a major public health priority as also dramatically formulated by the World Health Organization (http://www.who.int/mediacentre/news/releases/2014/amr-report/en/). The media report cases of patients dying in local hospitals from hitherto relatively harmless pathogens if they turn out to be antibiotic resistant and drastic surgical interventions remain the only option. Therefore, ‘Alternatives to Antibiotics’ became research calls of major grant organizations and the title of international conferences.

In January 2017, the US Food and Drug Administration (FDA) set new rules (https://www.fda.gov/AnimalVeterinary/GuidanceComplianceEnforcement/GuidanceforIndustry/ucm216939.htm) to limit antibiotic use in farm animals. FDA now bans the use of ‘medically important’ antibiotics (which are also used in humans) as antibiotic growth promoters (AGP) in animal husbandry. FDA has also launched discussions to change the status of antibiotics for disease prevention in farm animals from an over‐the‐counter to a veterinary prescription status. Until recently, a farmer could buy a 50‐kg bag of antibiotics in a veterinary feed supply store without a prescription while a personal use of 200 g antibiotic needed a prescription by a doctor. According to a recent FDA report, 62 per cent of antibiotics given to animals in 2015 were medically important for human health (https://www.fda.gov/downloads/ForIndustry/UserFees/AnimalDrugUserFeeActADUFA/UCM534243.pdf?source=govdelivery&utm_medium=email&utm_source=govdelivery) and could thus contribute to the selection of antibiotic‐resistant pathogens particularly when used at subtherapeutic doses. The amounts of antibiotics used in agriculture are huge: 17 million kg of antibiotics were used in farm animals compared to an estimated 4 million kg in human per year in the USA. A major part of these antibiotics is given in small doses to healthy chicken, cattle and pigs to accelerate weight gain, or in slightly larger doses (but still smaller than needed for treating an infection) to healthy animals for preventing infections. With the ban on antibiotics as growth promoters and the proposed regulation to put disease prevention under prescription, the FDA hopes to see less selection pressure for pathogens to develop antibiotic resistance. It is currently not clear whether the FDA ban is binding or only a recommendation. In the USA, change might also be encouraged by consumer groups who put pressure on fast food restaurants to use meat from chicken raised with minimal or no antibiotics and who publish reports about compliance by the different companies.

The US in‐feed antibiotics ban creates also new business opportunities for the animal feed industry and job opportunities for microbiologists. As discussed at the second conference on ‘Alternatives to Antibiotics’ ‘ATA2’ jointly organized in December 2016 by The World Organization of Animal Health (OIE) and by the US Department of Agriculture and formulated in a conference report, microbiome research could play an important role in business development (https://www.ars.usda.gov/alternativestoantibiotics/Symposium2016/index.html). In the animal feed industry, nutritional supplements is a very active and growing field, many new products were launched, and these activities have created an increasing demand for microbiologists to develop alternative growth promoters for farm animals. One approach would be to study the impact of in‐feed antibiotics used at subtherapeutic (subinhibitory) concentrations on the gut microbiome of farm animals (Looft and Allen, 2012; Brüssow, 2015). Some literature data suggest positive nutritional effects of antibiotics; for example, monesin and related antibiotics induce a microbiota change in ruminants that favours the production of propionate, reduces the production of methane and increases feed efficiency. Once gut microbes associated with growth promotion and growth inhibition are identified, specific stimulatory and inhibitory compounds modulating the gut microbiome could be developed based on this knowledge. Microbiome research showed that in‐feed antibiotics (chlortetracycline, sulfamethazine, penicillin) and parenteral amoxicillin led in pigs to a marked increase in *E. coli* in the gut microbiota. Carbadox antibiotic, another common AGP in pig rearing, caused a relative increase in *Prevotella* and abrogated an *E. coli* population shift during dietary change. Tylosin accelerated the maturation of the swine gut microbiota rather than altering its composition. In‐feed antibiotics in experimentally exposed chicken showed mainly changes in gut lactobacilli. Drawing a consensus from the observations is at the moment difficult because gut microbiome effects vary with animal species, antibiotic class and antibiotic doses. Where weight development was assessed in parallel, no significant effects were observed. Some scientists concluded from the observations that most growth promoting antibiotic effects occur indirectly via prevention of infections while metabolic effects on the host cannot be excluded.

Particularly, probiotic development for animal nutrition is a very active field. Two target areas can be identified: on one hand, probiotics use to re‐establish a healthy gut commensal microbiota disturbed by an oral antibiotic treatment (probiotics as antibiotic adjunct in disease treatment) and on the other hand probiotics that reduce the pathogenicity of opportunistic infectious agents (probiotics as an antibiotic alternative in disease prevention). As the animal feed industry in Europe cannot claim for probiotics antimicrobial effects against pathogens – such products would fall under drug regulation – probiotics are currently marketed for growth‐enhancing benefits and as ‘gut flora stabilizer’. Adisseo, number three of the animal feed industry, has just launched a *Bacillus subtilis* probiotic product, which was co‐developed with Novozymes, a global biotechnology company specialized in enzymes. Motivated by the explosion of the microbiome literature, Novozymes is now also increasingly interested in microorganisms. Chr. Hansen – another global company selling starters and health beneficial bacterial cultures, probiotics and enzymes for food and feed industries – has launched probiotics as feed supplements for poultry targeting *Clostridium perfringens*‐associated necrotic enteritis and recently probiotics against pathogenic *E. coli* infections in piglets. Companies like Evonik, specialist in feed amino acids, are now diversifying into the probiotic area for sustainable and antibiotics‐free livestock management. Evonik has a *Bacillus amyloliquefaciens* for poultry and *Enterococcus faecium* for piglets in its portfolio and is about to launch a third direct fed probiotic. DSM, a science‐based Dutch company active in health, nutrition, and materials, sells a probiotic *E. faecium* strain ‘to stabilize gut microflora’. In addition, Evonik and DSM have recently joined force to produce omega‐3 fatty acids from algae for a sustainable aquaculture. Altogether, research and development around these efforts to supplement feeds with probiotics are increasingly translating into job opportunities for microbiologists. Probiotics are also discussed for aquaculture and may be particularly relevant for the shrimp production industry in Asia, which currently relies on high amounts of antibiotics. At the ATA2 meeting in Paris, many Asian participants were present with a particularly high share of Chinese scientists both from the academic and company sector demonstrating their interest in antibiotic‐free animal feed.

Feed supplements are not limited to probiotics and prebiotics, other compounds are increasingly explored for growth promotion, particularly formic acid in protonated form as an ‘acidifier’ of the intestinal tract in chicken possibly reducing diarrhoea impact. Also chemical companies like BASF are active in that field investigating short‐chain fatty acids for nutrition and gut microbiota modulation. In chicken, formic acid reduced *Campylobacter jejuni* colonization and increased clostridia in the crop without lowering the pH of the intestinal digesta. A blend of organic acids and essential oils stimulated body weight gain and decreased *E. coli*, while lactic acid increased *Lactobacillus* in the chick ileum.

Beyond the animal feed supplement field, microbes and microbial compounds are investigated as antimicrobial agents. These approaches include bacteriophages, phage lysins and bacteriocins that directly kill bacterial pathogens by viral infection (phages), enzymatic action (phage lysins) or lytic action (bacteriocins). As treatment and prevention of bacterial infections with these products would represent medical activities, they will fall under drug legislation and need rigorous proof of efficacy, hence substantial financial investments including further job opportunities for microbiologists. As clinical trials have not yet provided a sound support for these concepts as antibiotic alternatives, investments are only increasing slowly and so far only small biotech companies are active in that medical field (e.g. Pherecydes from France in phage therapy or ContraFect in the US developing phage lysins for medical use). Job opportunities will increase when the first successful clinical trials with phage products are published. Notably, a recent controlled clinical trial with phage therapy underlined the need for microbiome research to identify suitable bacterial target pathogens for phage therapy (Sarker *et al*., 2016). Another potential application of phages could be their combination with antibiotics. Phage‐antibiotic synergy has been reported where the treatment with both agents yielded results superior to the sum of their individual actions (Oechslin *et al*., 2017). An added major advantage of the combined treatment approach could also be an inhibition of resistance development as pathogens would have to develop resistance both to antibiotics and to phages, which is probably more difficult to achieve than against each individual agent. Well‐designed laboratory experiments have shown that in confined environments phages could even impose a return to antibiotic sensitivity in initially mostly antibiotic‐resistant bacteria. It was argued that such combined phage/antibiotic application could prolong the time antibiotics remain clinically useful. As phage's therapeutic efficacy against the pathogen would not need to be claimed for this activity, reducing the level of resistance development against the antibiotic might not fall under drug legislation, and this may motivate non‐pharmaceutical companies into this type of phage development. Some companies have already developed phage approaches for food safety and food factory sanitation and received FDA approval for this type of phage use (e.g. Intralytix in USA) or with phage lysins in cosmetic products for skin conditions including eczema, acne and psoriasis like ‘Gladskin’ from the Dutch biotech company Micreos. With nisin, bacteriocins have reached the market place with commercial products for antimicrobial preservatives active against food spoilage organisms and food pathogens. DuPont Danisco is here the market leader followed by Royal DSM, both companies run research laboratories with microbiologists.

Instead of targeting pathogens with antimicrobial agents, there might also be possibilities to develop probiotic bacteria as ‘gut microbiota stabilizers’ in humans. Such products could be specifically designed for antibiotic prescription, which frequently lead to commensal gut microbiota disturbances and manifest as antibiotic‐associated diarrhoea (AAD). Meta‐analysis suggests that the consumption of specific probiotic strains is associated with lower rates of AAD in children and adults, but not in elderly patients. The studies were of heterogeneous quality, differed for antibiotic type used and probiotic applied. Most studies in that field relied only on a handful of probiotic bacteria, mainly lactobacilli (*L. rhamnosus* GG, *L. casei* Shirota, *L. acidophilus*), different bifidobacteria or the yeast *Saccharomyces boulardii*. These probiotics generally do not re‐colonize the disturbed gut microbiome, but were designed to restore the physiological gut microbiome composition. The mechanism of probiotic action is frequently not clear and defining these processes with microbiome research could lead to ameliorated probiotic strains. Empirically, doctors in some European countries prescribe an *Enterococcus faecium* probiotic as adjunct to antibiotic treatment to prevent gut microbiome disturbances. Following the success of faecal transplantation in *Clostridium difficile* patients and building on earlier clinical trials with synthetic defined bacterial strain mixtures, bacterial cocktails for re‐colonization of a gut microbiome disturbed by intensive antibiotic treatment like in *C. difficile* infections are intensively explored. In fact, oral antibiotic treatment has not only disturbing effects on the gut, but also on other body sites like the vaginal microbiome which can potentially also be alleviated with probiotic approaches. The current trends in microbiome research reinforce the high potential of microbiological research for translation into practical application leading to new companies, extended activities in existing companies and job creation for microbiologists mastering microbiome research as well as classical microbiology. Bacteriology has thus maintained its eminent role as both a basic and applied branch of science, with important impacts on medicine and industry as predicted in a now 100‐year‐old editorial announcing the genesis of a new science (Sedgwick, 1916).

## Conflict of interest

None declared.
